# The complement alternative pathway in paroxysmal nocturnal hemoglobinuria: From a pathogenic mechanism to a therapeutic target

**DOI:** 10.1111/imr.13137

**Published:** 2022-09-15

**Authors:** Antonio M. Risitano, Camilla Frieri, Eleonora Urciuoli, Luana Marano

**Affiliations:** ^1^ AORN San Giuseppe Moscati Avellino Italy; ^2^ Federico II University of Naples Naples Italy; ^3^ Severe Aplastic Anemia Working Party of the European Society for Blood and Marrow Transplantation Leiden Netherlands

**Keywords:** alternative pathway, C3, extravascular hemolysis, FB, FD, intravascular hemolysis, paroxysmal nocturnal hemoglobinuria

## Abstract

Paroxysmal nocturnal hemoglobinuria (PNH) is a rare clonal, not malignant, hematological disease characterized by intravascular hemolysis, thrombophilia and bone marrow failure. While this latter presentation is due to a T‐cell mediated auto‐immune disorder resembling acquired aplastic anemia, the first two clinical presentations are largely driven by the complement pathway. Indeed, PNH is characterized by a broad impairment of complement regulation on affected cells, which is due to the lack of the complement regulators CD55 and CD59. The deficiency of these two proteins from PNH blood cells is due to the somatic mutation in the phosphatidylinositol N‐acetylglucosaminyltransferase subunit A gene causing the disease, which impairs the surface expression of all proteins linked via the glycosylphosphatidylinositol anchor. The lack of the complement regulators CD55 and CD59 on PNH erythrocytes accounts for the hallmark of PNH, which is the chronic, complement‐mediated intravascular hemolysis. This hemolysis results from the impaired regulation of the alternative pathway upstream in the complement cascade, as well as of the downstream terminal pathway. PNH represented the first indication for the development of anti‐complement agents, and the therapeutic interception of the complement cascade at the level of C5 led to remarkable changes in the natural history of the disease. Nevertheless, the clinical use of an inhibitor of the terminal pathway highlighted the broader derangement of complement regulation in PNH, shedding light on the pivotal role of the complement alternative pathway. Here we review the current understanding of the role of the alternative pathway in PNH, including the emergence of C3‐mediated extravascular hemolysis in PNH patients on anti‐C5 therapies. These observations provide the rationale for the development of novel complement inhibitors for the treatment of PNH. Recent preclinical and clinical data on proximal complement inhibitors intercepting the alternative pathway with the aim of improving the treatment of PNH are discussed, together with their clinical implications which are animating a lively debate in the scientific community.

## 1 INTRODUCTION

Paroxysmal nocturnal hemoglobinuria (PNH) is a rare clonal, not malignant, hematological disorder characterized by intravascular hemolysis, severe thrombophilia, and bone marrow failure.^1^, ^2^ Even if bone marrow failure is not clinically evident in all patients,^3^ a T‐cell mediated auto‐immune attack of hematopoiesis, resembling that usually seen in idiopathic acquired aplastic anemia, is considered one of the key pathogenic events causing the disease. Indeed, for PNH a dual pathophysiology has been postulated: (a) the occurrence of a somatic mutation in the phosphatidylinositol N‐acetylglucosaminyltransferase subunit A (*PIGA*) gene in one or more hematopoietic stem cells (HSCs)^4^, ^5^, ^6^; and (b) the clonal expansion of these *PIGA*‐mutated HSCs, due to an immune escape from the T‐cell mediated autoimmune attack destroying normal (*PIGA*‐unmutated) hematopoietic progenitors.^7^, ^8^, ^9^ The molecular mechanisms by which the *PIGA* mutation leads to such immune escape have not been fully elucidated, and different hypotheses have been postulated,^1^ starting with the possibility that the *PIGA* mutation may affect the surface expression of the antigens putatively causing aplastic anemia.^8^, ^10^ On the other hand, the pathogenic role of *PIGA* mutations in other PNH manifestations is quite established, since these mutations impair the biosynthesis of the glycosylphosphatidylinositol (GPI) anchor.^11^ As a consequence, affected progeny mature blood cells lack from their surface all so‐called GPI‐linked proteins.^11^, ^12^, ^13^, ^14^ Among these missing proteins, there are also the two redundant complement inhibitors CD55 and CD59; as a consequence, PNH erythrocytes are unable to modulate complement activation on their surface, eventually accounting for the complement‐mediated intravascular hemolysis which is the hallmark of PNH.^1^ This impaired complement regulation seems also involved in the thrombophilic status typical of PNH, both directly through functional changes of PNH platelets, and indirectly through the pro‐thrombotic effect resulting from intravascular hemolysis.^15^ Here we review at the molecular level the derangement of the complement cascade in PNH, focusing on the key role of the alternative pathway, which recently emerged as an ideal therapeutic target for PNH treatment. Cutting‐edge clinical data with alternative pathway inhibitors will also be reviewed, with the aim of discussing pros and cons of different strategies of therapeutic complement inhibition in PNH.

## 2 THE ROLE OF THE ALTERNATIVE PATHWAY IN THE PATHOPHYSIOLOGY OF PNH

As stated above, the dysregulation of the complement cascade in PNH is due to the lack of CD55 and CD59 from the surface of affected erythrocytes (leading to hemolysis, and indirectly to thrombosis), and possibly of affected platelets or even neutrophils (likely contributing to thrombosis).^15^ But complement, and even complement regulation, is more than CD55 and CD59, and these two molecules need to be discussed in a broader context. Indeed, the complement system^16^, ^17^ should not be considered just as an ancient and primitive mechanism of the innate immunity that contributes to the clearance of microbes and of other foreign intruders. Our current understanding of the complement implies a broader role of this pathway within the immune system and inflammation,^18^ which includes a key function in immune surveillance against not only microbes and other foreign intruders, but also damaged host tissues (such as cellular debris and apoptotic cells possibly generating due to ageing or malignant transformation).^19^ Furthermore, the complement system is by‐directionally embedded with the inflammation, since inflammatory cells and cytokines may activate the complement cascade, and vice versa the complement anaphylatoxins may promote inflammation, creating an amplification loop which emerged once more in the recent description of COVID‐19 pathophysiology.^20^ Thus it is not surprising that its pivotal role in tuning both innate and adaptive immune response evolved together with its fine regulatory role, and that impairment of such regulation is implicated in the pathophysiology of several human diseases.^21^

In the context of impaired complement regulation which may lead to human disease, we have PNH, and the GPI‐linked proteins CD55 and CD59. CD55 and CD59 are just two of the several endogenous complement regulators, which also include two additional surface proteins (complement receptor 1 – CR1 or CD35, and membrane cofactor protein – MCP or CD46), and the two fluid‐phase proteins complement factor I – FI, and complement factor H – FH.^22^ All together, they modulate the activity of the complement alternative pathway (CAP), which included as its key components complement factor D (FD), complement factor B (FB), properdin (P), and of course the central complement component 3 (C3).^22^ CAP is activated by different triggers, mostly exogenous; but in contrast to complement classical and mannose/lectin pathways (CCP and CMP, respectively), the CAP is also characterized by a continuous, low‐grade activation due to the spontaneous hydrolysis of the internal thioester bond of complement component 3 (the so called “C3 tick‐over”).^23^ This reaction generates C3H_2_O, a C3b‐like molecule which is able to recruit factor B leading to a fluid phase pro‐C3 convertase. Subsequently, this pro‐convertase may be modified by the cleavage of FB by FD, eventually forming the C3 convertase C3H_2_OBb, which then generates C3b molecules that lead to the formation of membrane‐bound (through glycophorin A) C3 convertases.^22^ The enzymatic nature of the C3 convertase, which may generate several C3b molecules, eventually leading to the generation of additional C3 convertases (and then C5 convertases, once multiple C3b molecules are incorporated in the complex), leads to the so‐called amplification loop of the complement cascade.^22^ Indeed, irrespective of the trigger initiating any of the complement pathway, this amplification results in the generation of downstream effector complement molecules, which includes the anaphylatoxins C3a and C5a, and mostly C5b, which may incorporate C6, C7, C8 and C9 eventually leading to the formation of the lytic Membrane Attack Complex (MAC).^22^

As stated above, CD55 and CD59 exert a key role in regulating different steps of this complicate sequence of enzymatic reactions. Indeed, CD55 (also known as Decay Accelerating Factor, DAF) affects the formation and the decay of the C3 convertase,^24^, ^25^, ^26^ thus disabling the upstream activation of the complement cascade. In contrast, CD59 (or Membrane Inhibitor of Reactive Lysis, MIRL) prevents the incorporation of C9 onto the C5b‐C8 complex,^27^ thus acting downstream cascade by disabling MAC formation.^28^, ^29^ The hierarchical contribution of CD55 and CD59 to hemolysis suggests that CD59 is the key molecule which, if absent, leads to lysis,^30^ as supported by the lack of hemolysis seen in subject with inherited CD55 deficiency (the so‐called Inab phenotype).^31^ This dominant effect of CD59 might also be due to still unknown molecular mechanisms,^32^ which may include a direct inhibitory effect on the generation of the C3 convertase, as recently suggested.^33^ In PNH, both CD55 and CD59 are lacking from the surface of affected erythrocytes; this lack account for the well‐known susceptibility of PNH erythrocytes to complement‐mediated lysis.

The intrinsic susceptibility of PNH erythrocytes to lysis was initially described by Dr. Ham, who showed that these cells may lyse in vitro once incubated in autologous serum in conditions which activate the complement system^34^: this led to the introduction of the Ham test, which remained the diagnostic assay of PNH for almost half century. Some decades later, a more detailed description of the sensitivity of PNH erythrocytes to complement‐mediated lysis was performed by Sir Dacie and Dr. Rosse, who also demonstrated the existence of different PNH phenotypes characterized by different susceptibility to lysis.^35^, ^36^ Indeed, they found that susceptibility to complement‐mediated lysis of erythrocytes from PNH patients may be severe (15–25 times the normal one), or just a moderate (3–5 times the normal one),^35^, ^36^ eventually prompting the subsequent identification by flow cytometry of possible complete or partial deficiency of GPI‐linked proteins (referred as type III and type II PNH phenotypes, respectively),^37^, ^38^ which may result from null and non‐null *PIGA* mutations.^39^ This in vitro susceptibility accounts for the typical hallmark of PNH in vivo, namely the intravascular, complement mediated hemolysis, which appears chronic (as spontaneous CAP activation is chronic), but usually includes possible acute exacerbation (at time of brisk complement activation, triggered by infectious event, for instance), with the typical paroxysms which give the name to the disease.^40^ Indeed, while the impairment of complement regulation remains the same on PNH erythrocytes, the extent of complement activation may largely vary over time due to a number of triggers (the so‐called Complement Amplifying Conditions, CAC), which eventually drive the disease course over time (at least in terms of hemolysis).

## 3 THE ROLE OF THE ALTERNATIVE PATHWAY IN THE CONTEXT OF STANDARD ANTI‐C5 TREATMENT OF PNH

Treatment of PNH was merely supportive until 2004, when the first complement inhibitor eculizumab was introduced in the clinic.^41^ Eculizumab is an anti‐C5 monoclonal antibody which binds to circulating C5,^42^, ^43^ thereby preventing its cleavage into C5a and C5b, thus disabling the downstream terminal complement. After a first pilot, proof‐of‐concept study showing efficacy in terms of inhibition of intravascular hemolysis,^44^ the efficacy of eculizumab was systematically investigated in two large phase III, placebo‐controlled, randomized studies in two different PNH populations. These two studies showed that eculizumab inhibits intravascular hemolysis of PNH, eventually leading to reduction (or even abolishment) of red blood cell transfusion and hemoglobin stabilization, as well as to resolution of most hemolysis‐related symptoms.^45^, ^46^ Eculizumab was also able to reduce the rate of thromboembolic events,^47^ which contributed to the remarkable survival rates which approach 95% at 5 years.^48^, ^49^ Based on these data, since about 15 years eculizumab is the well‐established standard care of PNH, with the only limitation represented by the reduced availability in some geographic areas due to its very high price.^50^

The tremendous efficacy of eculizumab, an anti‐C5 agent, as treatment of PNH led to the misconception that PNH is a terminal complement‐mediated disease; this may appear in contrast with the pathogenic mechanisms described above, which rather imply a key role for the alternative pathway. Our group systematically investigated PNH biology during eculizumab treatment, demonstrating that anti‐C5 treatment may rather contribute to better understand the role of different pathways of the complement cascade in PNH pathophysiology.^51^ We documented that the pharmacological blockade of C5 may unmask the uncontrolled activity of the alternative pathway on PNH erythrocytes, which eventually may lead to accumulation of C3^52^, ^53^ (and of its split fragments)^54^ on PNH erythrocytes surface. Indeed, when erythrocytes of PNH patients were systematically and longitudinally analyzed by flow cytometry, we observed that all patients on eculizumab harbor a clear‐cut population of PNH erythrocytes (but not normal erythrocytes, based on CD59 expression) which bind C3 fragments (mostly C3d).^52^, ^53^ This finding does not have to surprise: indeed, on eculizumab PNH erythrocytes are spared from MAC‐mediated intravascular hemolysis, but continue to suffer from continuous early complement activation due to uncontrolled C3‐convertase activity (due to the lack of CD55, and possibly CD59^33^). In other words, functionally speaking, eculizumab may replace CD59 (which is missing on PNH), but the lack of CD55 ineluctably leads to surface C3 activation on PNH erythrocytes, once a threshold level of complement activation is reached. This is confirmed by in vitro data, showing that all PNH erythrocytes may eventually become C3 opsonized if exposed to brisk complement activation (ie, triggered by mild acidification) in presence of eculizumab.^55^ Notably, these PNH erythrocytes initially show C3b on their surface, but then this is quickly converted into its inactive split fragment C3d, which looses its enzymatic activity within C3/C5 convertases.^54^ These data highlight the broad derangement of complement regulation on PNH blood cells, which included uncontrolled activity of the C3 convertase (of any complement pathway) leading to C3 opsonization of PNH erythrocytes in vivo.^52^, ^53^

The observation of C3 opsonization of PNH erythrocytes during eculizumab has remarkable clinical implications; indeed, we have documented that these erythrocytes have a shorter half‐life in vivo,^53^ confirming that they can be removed by professional macrophages through the C3 cognate receptor complement receptor 3.^56^ In contrast to in vitro data,^55^ in vivo the proportion of PNH erythrocytes that appear opsonized with C3 fragments is extremely heterogeneous among patients^53^; genetic polymorphisms of endogenous complement regulators may contribute to this heterogeneity, as already demonstrated for the complement receptor 1 (its hypomorphic variant is associated to increased C3 opsonization, both in vitro and in vivo).^57^ Notably, C3 opsonization is associated with residual anemia, increased biomarkers of hemolysis (such as reticulocyte count and bilirubin)^53^; this led to the description of C3‐mediated extravascular hemolysis as an emerging mechanism of disease in PNH patients on eculizumab.^51^, ^58^, ^59^ Even if initially some PNH experts underestimated the importance of this novel mechanism of disease,^60^ now C3‐mediated extravascular hemolysis was confirmed by several groups,^53^, ^61^, ^62^ and it represent a key factor in driving the hematological benefit of PNH patients on eculizumab treatment.^63^

Nevertheless, we have to acknowledge that the degree of C3‐mediated extravascular hemolysis is very heterogeneous among patients, as are their hemoglobin levels, which also depend on residual intravascular hemolysis and compensatory erythropoiesis.^51^, ^58^, ^64^ Last but not least, it has to be emphasized that C3‐mediated extravascular hemolysis should not be considered a possible complication emerging upon treatment with eculizumab (or any anti‐C5) but rather an unavoidable phenomenon that is unmasked when the lytic terminal complement is pharmacologically disabled, and the broader impairment of complement regulation of PNH erythrocytes becomes clinically detectable.

## 4 THE ALTERNATIVE PATHWAY AS POSSIBLE THERAPEUTIC TARGET IN PNH

We have recently tried to better assess the hematological benefit during eculizumab treatment; indeed, registration studies did not include hemoglobin as an endpoint, and systematic data on hemoglobin response are lacking. We have first reported that residual anemia was quite frequent in PNH patients on eculizumab^53^; more recently, we tried to define detailed response categories.^63^ These categories seem to intercept meaningful clinical scenarios: indeed, in a recent multinational cohort we found that only about 15% of PNH patients may achieve normal hemoglobin on eculizumab treatment.^65^ Thus, residual anemia may emerge as an unmet clinical need in PNH, and alternative treatment strategies would be welcome; and novel targeted therapies should take in account what we have learnt from eculizumab treatment.^51^, ^58^ Based on the pathogenic mechanisms described above, including C3‐mediated extravascular hemolysis triggered by the alternative pathway, in 2009 we hypothesized that a more rationale anti‐complement treatment for PNH should intercept the complement cascade more proximally, at the level of the alternative pathway instead of the terminal pathway.^53^, ^59^ Since then, this interesting idea^51^, ^64^, ^66^ progressively developed involving several academic groups and pharmaceutical companies. Thanks to this tremendous collaborative work now we have a new class of complement‐modulating agents called “proximal complement inhibitors” which are in preclinical or clinical development for the treatment of PNH.^63^, ^67^, ^68^ Here we review the most relevant agents and data, focusing on the ones which target the alternative pathway, since this is the most relevant one in PNH pathophysiology.

## 5 TREATMENT STRATEGIES TARGETING THE ALTERNATIVE PATHWAY: PRECLINICAL WORK

**FIGURE 1:**
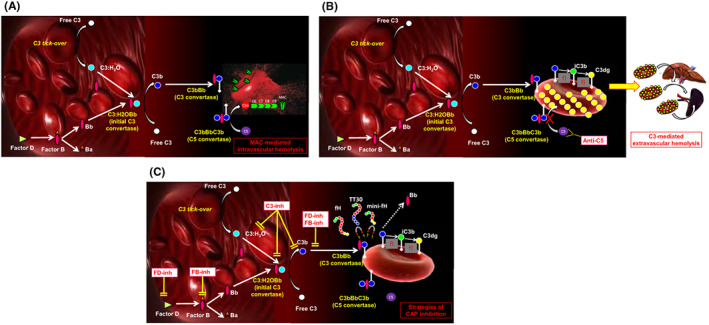
Complement alternative pathway in PNH. (A), Untreated PNH. C3:H_2_O generated by spontaneous hydrolysis of C3 (the so‐called “C3 tick‐over”) continuously initiates the complement cascade through its alternative pathway in the fluid phase. Due to the lack of CD55, PNH erythrocytes are unable to regulate complement activation on their surface, and C3bBb C3 convertase can be generated from C3 tick‐over and factor B cleavage operated by factor D. These C3 convertases generate further C3b, eventually self‐transforming into the C3bBbC3b C5 convertases, which cleave C5 into C5a and C5b. This latter may start the formation of the membrane attack complex with C6, C7, C8 and C9, eventually leading to intravascular hemolysis. (B), PNH on C5‐inhibitors. Terminal complement inhibitors (ie, anti‐C5 agents) prevent the cleavage of C5 into C5a and C5b, thereby disabling the formation of the membrane attack complex (MAC) and inhibiting intravascular lysis of PNH erythrocytes. Nevertheless, early steps of complement activation and upstream C5 cleavage remain uncontrolled, leading to opsonization of PNH erythrocytes with C3 fragments. C3‐opsonized erythrocytes can be recognized by C3‐specific receptors, expressed on professional macrophages in the liver and in the spleen, eventually resulting in extravascular hemolysis. (C), PNH on alternative pathway inhibitors. The alternative pathway may be intercepted at the level of different key components, such as FD, FB, C3 and C3 convertases. Inhibitors of any of these target, if the inhibition is pharmacologically sustained, disable the complement cascade in its early phases (these agents are also known as proximal complement inhibitors), preventing the generation of C3(H_2_O) Bb in the fluid phase, as well as disabling surface amplification activity acting on surface‐bound C3bBb. On PNH erythrocytes, this combined effect results in inhibition of the MAC‐mediated intravascular hemolysis, and in the prevention of C3 opsonization (and thus of extravascular hemolysis), eventually leading to normal life‐span of affected erythrocytes

As discussed above, the complement alternative pathway includes both activating proteins and negative regulators, which may all serve to develop strategies of complement inhibition or modulation. Indeed, activating proteins may serve as direct therapeutic targets, while endogenous regulators may be useful to develop engineered, recombinant regulators with inhibitory effect on the complement alternative pathway (Figure 1). We start describing these latter compounds, before introducing more traditional inhibitors of specific components of the complement alternative pathway.

### 5.1 C3 convertase‐targeting strategies: FH‐based engineered proteins

**FIGURE 2:**
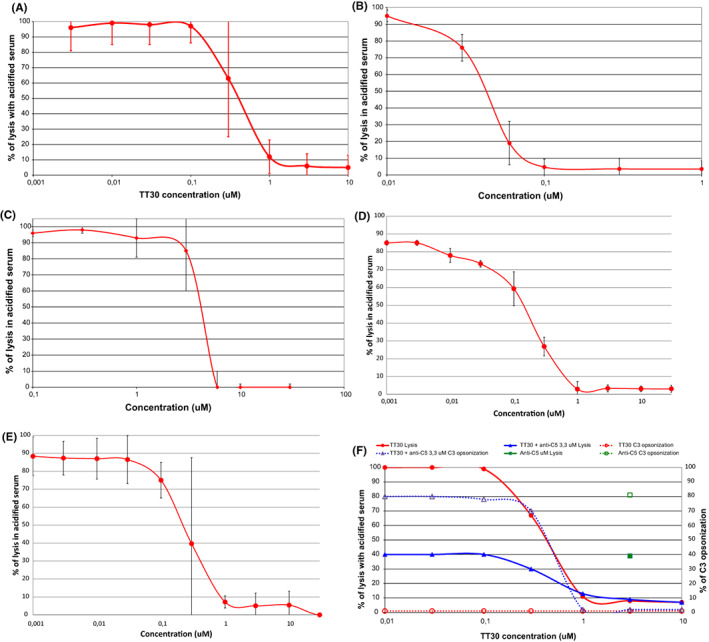
Dose–response curve of different alternative pathway inhibitors. (A), TT30. Effect of TT30 on lysis of PNH erythrocytes in vitro.^108^ (B), Mini‐FH. Effect of mini‐FH on lysis of PNH erythrocytes in vitro.^72^ (C), Compstatin. Effect of the compstatin analogue Cp40 on lysis of PNH erythrocytes in vitro.^80^ (D), FD inhibitor. Effect of the FD inhibitor “compound 7” on lysis of PNH erythrocytes in vitro.^85^ (E), FB inhibitor. Effect of the FB inhibitor LNP023/iptacopan on lysis of PNH erythrocytes in vitro.^86^ (F), Proximal inhibitor combined with anti‐C5. Effect of combination treatment including the proximal inhibitor TT30 and the anti‐C5 eculizumab (at fixed dose, 3.3 μM); effect on C3 opsonization is shown in addition to effect on lysis. In presence of anti‐C5, the contribution of the proximal inhibitors is better seen as inhibition of C3 opsonization, which becomes evident at doses overlapping to those of the dose–response in absence of anti‐C5 (see panel A of this figure). At the same concentration, the additive (and not synergic) effect on lysis also appears, leading to full inhibition of lysis (while at suboptimal concentration the effect on lysis was entirely driven by the anti‐C5)

This class of compounds was developed with the aim of generating engineered proteins which may recapitulate, and possibly improve, the modulatory effect of endogenous regulators of complement activation.^21^ Indeed, instead of using unmodified recombinant proteins, different scientists tried to modify functional domains of complement regulators with the aim of obtain a better inhibitory effect. In this direction, one of the strategies investigated the possibility of targeting such inhibitory domains at sites of complement activation, using the activated C3 split fragment C3b, or even further split fragments as iC3b/C3dg as a target.^69^ This goal was pursued by merging the functional domain of FH with a structure which may recognize C3, such as the complement receptor 2 (CR2). The first FH‐based fusion protein was developed in the laboratories of Prof Mike Holers, who invented TT30, a 65‐kDa engineered protein consisting of the iC3b/C3dg‐binding domain of CR2 merged with the functional domain of FH.^70^ Since TT30 recapitulated the modulatory effect of endogenous FH, it inhibits the complement alternative pathway both impairing the stability of the C3 convertase, and, as co‐factor of FI, by inactivating C3b into iC3b/C3dg (thus generating its own molecular target).^21^ Our group collaborated to the preclinical development of this compound generating an in vitro model of PNH^54^ which can be exploited to investigate the modulatory effect of any complement inhibitor on both lysis of PNH erythrocytes, and their opsonization by C3 fragments (looking for potential effect on extravascular hemolysis).^50^ Indeed, we demonstrated that TT30 fully prevented the lysis of PNH erythrocytes in vitro upon complement activation, and surviving erythrocytes (in contrast to what is seen with eculizumab) remain free from C3 fragments on their surface (Figure 2A).^54^ Based on these data which may support the concept that T30 may prevent C3‐mediated extravascular hemolysis, a phase I, single ascending dose trial in untreated PNH patients was started. Unfortunately, limited data are available from this trial, since they were reported just as a poster at the American Society of Hematology.^71^ TT30 was safe and well tolerated in the 10 patients enrolled in the study, pharmacokinetic and pharmacodynamics data showed that biological efficacy could be seen even after single dosing, but the inhibition was transient due short half‐life of the compound.^71^ As a consequence, the sponsor of the study decided to discontinue the clinical program. Nevertheless, this remains the first inhibitor of the complement alternative pathway (ie, upstream the terminal pathway) investigated in humans, as well as the first proof‐of‐concept of potential efficacy of complement alternative pathway inhibitors in PNH, even in monotherapy.

A second FH‐based complement modulator was developed in the laboratories of Prof John Lambris, who created an engineered fusion protein merging again the inhibitory domain of FH (and in particular its complement control protein [CCP] 1–4 domain) with its own C3‐binding domain CCP 19–20.^72^ The result is a small (43 kDa), miniaturized version of FH (ie, mini‐FH, also known as AMY‐201; Amyndas Pharmaceuticals), that we investigated in vitro exploiting our model of PNH. As TT30, in vitro mini‐FH was able to completely prevent the lysis of PNH erythrocytes, and their C3 decoration, with a dose–response curve even more favorable than TT30 (Figure 2B).^72^ Clinical translation programs with mini‐FH have not started yet, albeit they have been hypothesized.^73^

### 5.2 C3‐targeting strategies

While the FH‐based engineered proteins affect C3 activation by tuning the activity of C3 convertase, targeting surface‐bound C3 split fragment, a different strategy of complement inhibition aims to inhibit C3 more directly binding it even in its native form circulating in the fluid phase. This strategy is considered quite challenging, due to the very high plasma level of C3 (approximately 1 mg/mL, equal to about 6 μM) and to its large size (183 kDa). Nevertheless, both monoclonal antibodies and small peptides have been developed, and preclinical data are available.

#### 5.2.1 Anti‐C3 monoclonal antibodies

Professor Ron Taylor and his lab have developed several different monoclonal antibodies binding to C3 in its native form, as well as to its different split fragments (eg, C3b or iC3b/C3dg). One of these antibodies (the anti‐C3b/iC3b mAb 3E7, and its humanized derivative H17; EluSys Therapeutics) is specific for C3b incorporated into the C3bBb C3 convertase, and thus represents a selective inhibitor of the alternative pathway potentially interesting for PNH. Indeed, similarly to FH‐based proteins, this mAb was shown to result in full inhibition of hemolysis and prevention of C3 opsonization on PNH erythrocytes in vitro.^74^ While these data represent a further proof‐of‐concept that intercepting the alternative pathway at the level of C3 is potentially effective in PNH, the clinical development of anti‐C3 mAb has not started yet, since derivatives without their Fc fragments are needed to avoid that complete mAbs may serve as further opsonins, eventually hindering any clinical efficacy.^75^

#### 5.2.2 C3‐targeting peptides: compstatins

An alternative to mAbs for the direct inhibition of C3 is represented by small peptides. This field was pioneered by Professor John Lambris, who identified compstatin, a 13‐residue disulfide‐bridged peptide which is specific for human C3 and C3b.^76^ Compstatin, and all its analogs subsequently generated,^77^, ^78^, ^79^ are broad C3 inhibitors which, in addition to the alternative pathway and its amplification loop, inhibit also the classical and the lectin/mannose complement pathways.^76^ We investigated the efficacy of compstatin (and in particular of the third generation compstatin analog Cp40/AMY‐101 and its pegylated derivative; Amyndas Pharmaceuticals) in our in vitro model of PNH, showing that both lysis of PNH erythrocytes and their C3 opsonization were completely prevented (Figure 2C).^80^ AMY‐101 is now in its clinical development in several complement‐mediated diseases, which include periodontal disease,^81^ COVID‐19,^82^ and possibly PNH.^83^ A pegylated version of a second generation compstatin (APL‐2, also known as pegcetacoplan) was developed by Apellis; even if in this case preclinical data are scarce^84^ (but eventually consistent with those of other compstatins), the clinical translation program was quite quick and effective.

### 5.3 Selective alternative pathway inhibitors: targeting FD, FB and properdin

More selective inhibitors of the complement alternative pathway can be obtained targeting the initiating event of this pathway before the C3 convertase is generated (Figure 1); the three candidate targets are FD, FB and properdin. The first FD antibody was a monoclonal antibody developed by Roche/Genentech. More recently, several small peptide inhibitors were developed by different companies. We challenged in our in vitro model a FD inhibitor developed by Novartis, which showed excellent efficacy in terms of inhibition of the lysis of PNH erythrocytes, as well as prevention of C3 opsonization (Figure 2D).^85^ Similar data were generated with another small peptide developed by Novartis (LNP023, also known as iptacopan), which instead is a selective inhibitor of FB: again using our robust in vitro model, we found that iptacopan fully inhibited the lysis of PNH erythrocytes, which remained free from any opsonization by C3 split fragments (Figure 2E).^86^ More recently, evidence of in vitro efficacy in PNH were generated also with a mAb specific for properdin.^87^

### 5.4 Take‐home messages from preclinical data with inhibitors of the complement alternative pathway

Robust preclinical data are available for many alternative pathway inhibitors exploiting different strategies of complement modulation. These data are very consistent, since irrespective of the specific molecular target and of the specific class of inhibitor, all compounds showed that: (a) full inhibition of the alternative pathway can be achieved; (b) upstream inhibition of the alternative pathway also disable the activation of the terminal pathway; (c) These results were observed as single‐agent exposure; (d) albeit head‐to‐head comparison among agents was not performed (but in some cases data were generated in the same lab exploiting the same assay), all the compounds seem equivalent and preclinical data do not support any preference for any target or inhibitor. Thus, moving from in vitro activity to in vivo efficacy, the two key questions are: (a) whether these data can be reproduced in vivo (in particular in terms of efficacy on intravascular hemolysis and extravascular hemolysis in case of monotherapy); (b) how pharmacokinetic and pharmacodynamic features of each inhibitor may affect their in vivo safety and efficacy.

## 6 INHIBITORS OF THE ALTERNATIVE PATHWAY: CLINICAL DATA IN PNH

There are different inhibitors of the complement alternative pathway which are currently in clinical investigation for the treatment of PNH (Table 1). They can be split according to their specific target, since they aim to block the enzymatic cascade at the level of its key element C3 (which is also shared with the classical and the lectin/mannose pathway), or more proximally at the level of the two initiating components FD and FB.

**TABLE 1:** Inhibitors of the complement alternative pathway in preclinical and clinical development for PNH

Target	Agent	Preclinical data	Clinical program	Clinical trial ID	Design	Patient population^a^	Study treatment	Results	Marketing authorization
C3 convertase	TT30	Yes^108^	Yes	NCT01335165	Phase I, open‐label, SAD	Untreated PNH	IV infusion, SC injection	Yes^71^	No
	Mini‐FH	Yes^72^	No	N.A.	N.A.	N.A.	N.A.	N.A.	N.A.
C3	Pegcetacoplan	Yes^84^	Yes	NCT02264639	Phase Ib, open‐label, MAD, POC	Poor responders PNH	Daily, SC infusions	Yes^89^	Yes
				NCT02588833	Phase Ib, open‐label, MAD, POC	Untreated PNH	Daily, SC infusions		
				NCT03531255	Phase III, open‐label, extension	PNH exposed to APL‐2	Daily, SC infusions		
				NCT03500549	Phase III, randomized vs ecu	Poor responders PNH	SC infusions, BIH	Yes^91^	
				NCT04085601	Phase III, open‐label	Untreated PNH	SC infusions, BIH	No	
	AMY‐101	Yes^80^	Yes	EudraCT: 2020–004408‐32	Phase II, open‐label, randomized	ARDS in COVID‐19	SC infusion, daily	Yes (in press)	No
Factor D	Danicopan (ALXN2040)	Yes^109^	Yes	NCT03053102	Phase Ib, open label, MD, POC	Untreated PNH	Orally, TID	Yes^95^	No
				NCT03181633	Phase II, open‐label, extension	PNH exposed to ACH‐4471	Orally, TID	Ongoing	
				NCT03472885	Phase II, open label, MD, POC	Poor responders PNH	Orally, TID	Yes^96^	
				NCT04469465	Phase III, randomized vs ecu	Phase III, randomized vs ecu	Orally, TID	Ongoing	
	Vemircopan (ALXN2050)	No	Yes	NCT04170023	Phase II, open label, POC	Danicopan‐treated PNH, poor‐responders to anti‐C5 and untreated PNH	Orally, BID	Ongoing	No
	BCX9930	No	Yes	NCT04330534	Phase I‐II, open‐label, MAD	Healthy volunteers, untreated PNH, poor responder PNH	Orally, BID	Yes^97^, ^98^, ^99^, ^100^	No
				NCT05116774	Phase II, randomized vs anti‐C5	Poor responder PNH	Orally, BID	No	
				NCT05116787	Phase II, randomized vs placebo	Untreated Phase II, open‐label, MAD PNH	Orally, BID	No	
Factor B	Iptacopan (LNP023)	Yes^86^	Yes	NCT03439839	Phase II, open‐label, MAD	Poor responder PNH	Orally, BID	Yes^101^	No
				NCT04820530	Phase II, open‐label, MAD	Untreated PNH	Orally, BID	Yes^102^	
				NCT04747613	Phase II, open‐label, extension study	Iptacopan‐treated PNH	Orally, BID	No	
				NCT04558918	Phase III, randomized vs eculizumab	Poor responder PNH	Orally, BID	No	
				NCT04820530	Phase III, open‐label	Untreated PNH	Orally, BID	No	
Properdin	N.A.	C3	Yes^87^	N.A.	N.A.	N.A.	N.A.	N.A.	N.A.

Abbreviations: BID, *bis in die* (twice a day); BIH, *bis in hebdomade* (twice a week); Ecu, eculizumab; IV, intravenous; MAD, multiple ascending doses; N.A., not applicable; POC, proof‐of‐concept; QOD, *quaque die* (once a day); SAD, single ascending dose; SC, subcutaneous; TID, *ter in die* (thrice a day).

^a^ Stable or poor response is intended to standard eculizumab treatment.

### 6.1 C3‐targeting therapy: pegcetacoplan

The pegylated compstatin derivative pegcetacoplan (ratio POT‐4:Peg 2:1) was initially investigated in two phase Ib studies including PNH patients naive to treatment, or receiving eculizumab. In the naive patients study (PADDOCK trial, n = 14), pegcetacoplan given in monotherapy at the subcutaneous daily infusion (180 or 270 mg) increased hemoglobin values from 8.4 g/dL (range, 5.5–11.0) at baseline to 11.0 g/dL (range, 7.4–13.5) at day 29, with hemoglobin normalization in 8 out 14 patients.^88^ At day 85 these improvements were confirmed, with just one red blood cell transfusion given during the treatment period, and in absence of thromboembolic events or clinically meaningful infectious complications.^88^ In the eculizumab‐treated study (PHAROAH trial, enrolling patients with inadequate hematological response) pegcetacoplan was given as add‐on therapy, at different doses. In patients receiving therapeutic dose (270 mg or 360 mg daily, as subcutaneous infusions), 5 out of 6 patients achieved meaningful hemoglobin improvements.^89^ Four of the 6 patients continued the treatment as monotherapy (ie, discontinuing eculizumab) up to 2 years, showing sustained hemoglobin response and other biomarkers of hemolysis (LDH, bilirubin and reticulocytes) within the normal range or slightly increased.^89^

Subsequently, pegcetacoplan was investigated in the large phase III open‐label, randomized, PEGASUS study, which enrolled PNH patients with inadequate response to eculizumab (ie, hemoglobin <10.5 g/dL). According to the study design, all patients received a 4‐week run‐in period of add‐on treatment with pegcetacoplan (needed for pharmacokinetic reasons), before being randomized to receive either pegcetacoplan (at the dose of 1080 mg, subcutaneously, bi‐weekly; n = 41) or eculizumab (n = 39) in monotherapy.^90^ At week 16, the primary endpoint of hemoglobin change from baseline was achieved: indeed, pegcetacoplan was superior to eculizumab, with an adjusted mean treatment difference of 3.84 g/dL (*P* < 0.0001; +2.37 ± 0.36 g/dL and − 1.47 ± 0.25 g/dL from baseline in pegcetacoplan and eculizumab arms, respectively).^91^ The rate of transfusion avoidance was 35/41 (85.4%) in pegcetacoplan arm and 6/39 (15.4%) in the eculizumab arm, and the proportions of patients achieving hemoglobin normalization were 34.1% and 0% in pegcetacoplan and eculizumab arms, respectively.^91^ The long‐term analysis at 48 weeks (in the open‐label part of the study) confirmed the hemoglobin gain in comparison to baseline in patients randomized to pegcetacoplan (+2.47 ± 1.72 g/dL; n = 33), and patients switching to open‐label pegcetacoplan after being initially randomized to eculizumab achieved a similar hemoglobin gain (+2.93 ± 2.09 g/dL, n = 30).^92^ Transfusion avoidance was overlapping between the two treatment arms (73% in the pegcetacoplan‐pegcetacoplan arm and 72% in the eculizumab‐pegcetacoplan arm).^92^ This remarkable effect was observed also for other secondary endpoints such as reticulocyte count and bilirubin, as well as the proportion of PNH (type II + type III) erythrocytes, which increased from 66.8% ± 26.5% at baseline to 93.9% ± 6.4% at week 16 in the pegcetacoplan arm, but decreased from 72.9% ± 25.8% at baseline to 62.6% ± 26.0% at week 16 in the eculizumab arm.^91^ As expected, at week 16 C3d‐positive erythrocytes were undetectable in the pegcetacoplan arm (0.2% ± 0.3% compared to 17.7% ± 13.5% at baseline), and unchanged in the eculizumab arm (16.9% ± 15.5% compared to19.8% ± 15.0% at baseline).^91^

The safety profile was quite acceptable, since the treatment was well tolerated, and fatal event occurred during the study. Injection site reaction was the most frequent Treatment Emergent Adverse Event (TEAE; 36.6% and 2.6% in pegcetacoplan and eculizumab arms, respectively), which remained self‐limiting in most cases; other common TEAE were diarrhea (22.0% and 2.6%), headache (7.3% and 23.1%), and fatigue (4.9% and 15.4%).^91^ Serious adverse events were observed in seven patients (17%) in the pegcetacoplan arm and six patients (15%) in the eculizumab arm. Hemolysis was the only recurrent serious adverse event, leading to treatment discontinuation in 3 patients within week 16.^91^ Ten additional patients discontinued pegcetacoplan switching back to eculizumab during the open‐label period of the study, due to breakthrough hemolysis (n = 3), pancytopenia/aplastic anemia (n = 2), hematological cancers (n = 2), mesenteric ischemia (n = 1), hypersensitivity pneumonia (n = 1), and fatal SARS‐CoV‐2 infection (n = 1).^92^ Looking specifically for breakthrough hemolysis, the cumulative discontinuation rate through the 48 week study period was 7.5%.^92^ Irrespective of treatment discontinuation, the rate of breakthrough hemolysis in the pegcetacoplan was 10% (n = 4) at week 14, compared to 23% (n = 9) in the eculizumab arm,^91^ even if it was suggested that the clinical severity of these events was quite different in the two treatment arms.^93^, ^94^ This apparent inconsistency between the excellent efficacy, in terms of control of intravascular hemolysis, and the possible increased risk of severe breakthrough hemolysis may be explained by a transient loss of full C3 inhibition, as discussed below. Thrombotic events were not observed in any of the treatment arm at week 16, while 2 thromboembolic events associated with CAC were recorded in the open‐label treatment period. Infectious events were recorded at week 16 in 12 patients (29%) in the pegcetacoplan arm and in 10 patients (26%) in the eculizumab arm, with no statistically significant difference between treatment arms.^91^

Thus, in conclusion the PEGASUS trial demonstrated that pegcetacoplan monotherapy improved hematological response in PNH patients with inadequate response to eculizumab. This effect is due to an effective inhibition of MAC‐mediated intravascular hemolysis, combined with the prevention of C3‐mediated extravascular hemolysis. Based on these results, pegcetacoplan was approved by the FDA for patients with PNH who are either treatment‐naive or switching from anti‐C5 monoclonal antibodies, and by EMA just for PNH patients who remain anemic after a minimum of 3 months of treatment with anti‐C5 agents.

### 6.2 FD‐targeting therapies: danicopan, vemircopan and BCX9930

There are at least three small‐molecule factor D inhibitors which are in their clinical development: danicopan, vemircopan and BCX9930.

Danicopan is the first‐in‐class, orally available, FD inhibitor, for whom data are quite mature from two open‐label, single‐arm, phase two studies.^95^, ^96^ The first study enrolled 10 untreated, hemolytic PNH patients who were exposed to danicopan in monotherapy at the initial oral dose of 100–150 mg thrice daily, with possible further step‐wise escalation up to 200 mg in case of residual uncontrolled hemolysis (according to per protocol pre‐defined criteria). The primary endpoint of the study was change in LDH level at day 28. All enrolled patients reached the primary endpoint at 28 days; two then discontinued the treatment, one for a serious adverse event (liver enzymes increase in concomitance of breakthrough hemolysis), and one for personal reasons (unrelated to safety).^95^ Danicopan resulted in a significant reduction of LDH level, which decreased from mean 5.7 times upper limit of normal (ULN) at baseline to mean 1.8 times ULN at day 28 (*P* < 0.001). This effect on intravascular hemolysis was retained throughout the entire treatment period (at day 84, LDH was 2.2 times ULN; *P* < 0.001). The effect of LDH was associated with significant improvement in hemoglobin level, with 1.1 g/dL and 1.7 g/dL increase from baseline 9.8 g/dL at day 28 and 84, respectively (both *P* < 0.005). C3d deposition on PNH erythrocytes remain negligible during the study, with some increase of the percentage of PNH erythrocytes (from mean 32% ± 24.6% to mean 56% ± 19.9% at day 84, *P* = 0.001).^95^ Most common adverse events were headache and upper respiratory tract infections.^95^ The second study was conducted in PNH patients with poor response to eculizumab (defined as transfusion‐dependency), who were exposed to danicopan as an add‐on treatment on top of Eculizumab, given orally at the dose of 100–200 thrice daily.^96^ Twelve patients were enrolled in the study and started danicopan treatment (10 at 100 mg every 8 hours, two at 150 mg); all but one completed the treatment (with 7 titrating the dose to 150 mg, and three further to 200 mg every 8 hours), since one patient discontinued danicopan after the second dose due to a serious adverse event (a complication of a pre‐existing cardiac morbidity, which was judged unlikely related to the investigational treatment). The primary endpoint of the study was hemoglobin change, which increased from 7.9 ± 1.4 g/dL at baseline to 10.3 ± 1.6 g/dL at week 24 (mean increase 2.4 g/dL; *P* = 0.0001). In the 24 weeks prior to danicopan, 10 patients received 31 transfusions (50 units) compared with 1 transfusion (2 units) in 1 patient during the 24‐week treatment period. During the 24 weeks of treatment, the transfusion requirement was one transfusion (two units, in concomitance of a CAC) in one patient, compared with 34 transfusions (58 units) given to 10 patients (one patient refused transfusion due to religious beliefs) in the 24 weeks prior enrolment.^96^ Consistent with its mechanism of action, danicopan reduced (without abolishing) the percentage of C3d‐positive erythrocytes from mean 30% at baseline, to mean 18% at week 24. In parallel, the proportion of PNH erythrocytes increased from mean 54% at baseline to mean 84% at week 24. Further changes in laboratory parameters included reduction of absolute reticulocyte count and of bilirubin, while LDH level remain unchanged (but it was already normal‐like at baseline).^96^ Danicopan was very well tolerated, with headache, cough and nasopharyngitis being the most common adverse events. These data demonstrate that danicopan in monotherapy inhibits intravascular hemolysis in untreated PNH patients, preventing the emergence of C3‐mediated intravascular hemolysis. Then, once given as add‐on treatment on top of eculizumab in PNH patients with poor response (meaning transfusion‐dependent), danicopan inhibits C3‐mediated extravascular hemolysis leading to improved hemoglobin level and drastically reduced transfusion requirement.^96^ Based on these results, a phase III randomized study is currently investigating danicopan as add‐on treatment on top of standard anti‐C5 treatment in PNH patients with residual anemia to eculizumab or ravulizumab.

As proof‐of‐concept, results from the phase II danicopan studies prove that therapeutic FD inhibition prevent C3‐mediated extravascular hemolysis, potentially inhibiting also MAC‐mediated intravascular hemolysis. Nevertheless, some observations suggest that in monotherapy, at least in some patients, the protection from intravascular hemolysis was not complete (as documented by granular data of LDH, hemoglobin and PNH clone size). As add‐on treatment, the protection from intravascular treatment is provided by the concomitant eculizumab, but still full prevention of (in this case) extravascular hemolysis is not achieved (as documented by granular data of hemoglobin, C3d‐opsonization and PNH clone size). These data suggest that in some patients therapeutic inhibition of FD remain pharmacologically partial or suboptimal, likely due to pharmacokinetic and pharmacodynamic features of danicopan, which might preclude its broader use in monotherapy. For these reasons, the same pharmaceutical company continued to develop other FD inhibitors in vitro; thus, a second‐generation FD (also known as vemircopan) was selected for clinical development based on its better pharmacokinetics and pharmacodynamics. A phase II study enrolling three different PNH patient populations is currently ongoing, and initial data are expected for the American Society of Hematology meeting 2022.

Another FD‐targeted therapeutic program is conducted by a different pharmaceutical company, which brought in the clinic BCX9930 within two phase II studies in untreated PNH patients or in PNH patients with inadequate response to C5 inhibitors. BCX9930 was given orally at the starting dose of 50, 200 or 400 mg, eventually escalating it to the target dose of 400 or 500 mg. In 9 untreated PNH patients who have completed 48 weeks of treatment (out of 10 enrolled), BCX9930 in monotherapy led to remarkable increase of hemoglobin level from mean 8.25 gr/dl at baseline to mean 12.00 gr/dL at week 48, with no need of transfusion during the treatment period.^97^, ^98^ This improvement was associated with significant increase of the percentage of PNH erythrocytes, which moved from mean 47% at baseline to mean 90% at week 48.^97^, ^98^ In 4 PNH patients with inadequate response to C5 inhibitors who have completed 48 weeks of treatment (out of 6 enrolled), BCX9930 given initially as add‐on and then as monotherapy (3 out 4 patients) increased hemoglobin level from mean 8.91 gr/dl at baseline to mean 11.60 gr/dL at week 48, with no need of transfusion during the treatment period.^99^, ^100^ The percentage of PNH erythrocytes also increased from mean 57% at baseline to mean 89% at week 48, while in the same period the percentage of C3d‐opsonized PNH erythrocytes decreased from mean 14.4% to mean 5.9%.^96^, ^97^ The safety profile was quite good, with headache and self‐limiting rush as the most common side effect, and breakthrough hemolysis in two of the 16 enrolled patients (16%).^96^, ^97^ While these preliminary data (presented just as meeting abstracts) clearly demonstrate the potential effect of BCX9930 in controlling both intravascular and extravascular hemolysis in PNH even in monotherapy, more detailed information are waited to better understand the depth of alternative pathway inhibition achieved with this agent.

### 6.3 FB‐targeting therapy: iptacopan

At the moment the only FB inhibitor which is in clinical development is iptacopan (previously known as LNP023), an oral, selective and potent first‐in‐class agent which was investigated in two open‐label phase II studies in PNH. The first study was conducted in PNH patients who were poor responders to standard anti‐C5 treatment; the study included two patient cohort, with different criteria of poor response (based on LDH and hemoglobin values).^101^ Iptacopan was initially given as add‐on therapy on top of eculizumab; the starting dose was 200 mg twice a day on cohort 1, while in cohort 2 patients started at 50 mg twice a day, with possible escalation in case of suboptimal response. The primary endpoint of the study was change of LDH level at 13 weeks; notably, once the primary endpoint was reached, patients were offered to continue the treatment within a long‐term extension study, in which the standard anti‐C5 treatment could be modified or even discontinued. Data are available just for cohort 1 of the study^101^; 10 patients were enrolled, all with meaningful residual intravascular hemolysis (LDH >1.5 times the ULN) and anemia (all of them received blood transfusions in the 12 months before enrollment, range 2–40). All patients received iptacopan as per protocol, with no treatment discontinuation; they all continued iptacopan treatment within the extension study, and 7 out 10 eventually discontinued eculizumab thus receiving iptacopan in monotherapy. The drug was very well tolerated; 3 serious adverse events were observed during the study (one during the screening period), but none of them was deemed related to the investigational treatment.^101^ The primary endpoint of the study was reached: indeed, LDH reduced from the mean 539 ± 263 IU/L at baseline to the 245 ± 54 IU/L at week 13 (*P* = 0.006), eventually demonstrating the additive effect of iptacopan in controlling intravascular hemolysis once added to standard eculizumab. The effect on intravascular hemolysis was associated with a remarkable hematological benefit, since no patient received any blood transfusion during the study period, and hemoglobin level increased from mean 9.77 ± 1.05 g/dL at baseline to 12.63 ± 1.85 g/dL at week 13 (*P* < 0.001). Notably, eight out of 10 patients achieved hemoglobin normalization (>12 g/dL), exhibiting full resolution of anemia.^101^ Significant improvements up to normalization were observed also in other biochemical markers of hemolysis, such as bilirubin and reticulocyte count. The possible effect on C3‐mediated extravascular hemolysis was investigated looking to C3d‐opsonization on PNH erythrocytes: while C3d‐opsonization was quite large at baseline (20.5% ± 15.4%), it became negligible after the 13 week treatment with iptacopan (0.18% ± 0.12%), eventually confirming the full abrogation of extravascular hemolysis. This was confirmed also by the remarkable increase of the PNH erythrocytes population, which increased from 37.7% ± 24.8% at baseline to 79.8% ± 18.5% at week 13 (*P* = 0.0002).^101^ After the initial 13 week treatment period, in 7 patients who achieved control of intravascular and extravascular the standard eculizumab treatment was stopped. In these 7 patients receiving iptacopan in monotherapy no breakthrough hemolysis event was observed, even in case of single missed doses. Notably, when key efficacy endpoints were compared between combination treatment (at week 13) and monotherapy (last visit before data lock) no difference was observed: LDH moved from 238% ± 47% to 250% ± 62% IU/L, hemoglobin from 13.30 ± 1.98 to 13.03 ± 1.73 g/dL, and PNH erythrocyte clone size from 77.9% ± 22.2% to 84.0% ± 22.8%.^101^ These data clearly documented that iptacopan treatment results in better control on MAC‐mediated intravascular hemolysis and full prevention of C3‐mediated extravascular hemolysis even when used in monotherapy in PNH patients.

The second study investigated iptacopan in untreated hemolytic PNH randomizing patients with overt hemolysis to receive different schedules of iptacopan in monotherapy: in cohort 1 patients received iptacopan at 25 mg twice a day for 4 weeks, followed by dose escalation to 100 mg twice a day up to 2 years, while in cohort 2 the initial dose was 50 mg, then escalated to 200 mg. Thirteen patients were enrolled, and the interim analysis done on LDH change at week 12 was performed on 12 evaluable patients (one patient discontinued iptacopan after 2 days due to headache, and was not evaluable for efficacy). All the 12 evaluable patients achieved the 60% reduction in LDH levels which was set as primary endpoint of the study; more in detail, LDH reduction was achieved as rapidly as 2 weeks after treatment starting, irrespective of the dose cohort, with mean reduction by 76.5% (90% CI: 90.1, 63.0; *P* < 0.0001) and 85.0% (90% CI: 92.8, 77.2, *P* < 0.0001) in cohorts 1 and 2, respectively.^102^ These results were sustained throughout the study, including long‐term extension; even if no patient exhibited LDH reduction <40% in any study cohort, patients in cohort 2 (the higher‐dose group) all showed sustained inhibition of intravascular hemolysis (defined as LDH <1.5 times the ULN), and less variability of LDH levels compared to lower‐dose cohort. As in the poor responder study, the hematological benefit was remarkable: all but one patient (who had a concomitant reticulocytopenia due to underlying myelodysplastic syndrome) did not receive any blood transfusion during the study. In addition, iptacopan led to significant improvement in hemoglobin level, which increased from mean 8.85 g/dL at baseline to mean 11.52 g/dL at week 12 in cohort 1, and from mean 7.69 g/dL at baseline to 10.90 at week 12 in cohort 2. Changes in other biomarkers of hemolysis such as bilirubine, reticulocyte count, haptoglobin were consistent with a remarkable control of MAC‐mediated intravascular hemolysis. Furthermore, the prevention of C3‐mediated intravascular hemolysis was documented by negligible C3d‐opsonization of PNH erythrocytes, and confirmed by the significant increase of PNH erythrocytes from mean 33.6% at baseline to mean 82.9% at week 12.^102^ Iptacopan treatment was safe and well tolerated, since no death and no serious adverse event was observed during the study. The most common AEs were headache (possibly related to the drug and its efficacy, as observed at the beginning of any effective anti‐complement treatment in PNH), abdominal discomfort, elevation of plasma alkaline phosphatase, cough, oropharyngeal pain, pyrexia, and upper respiratory tract infection. Notably, no episode of breakthrough hemolysis was observed during the study, nor any thromboembolic event. These data clearly confirm that iptacopan results in full inhibition of intravascular hemolysis in untreated PNH patients, and prevent the emergence of extravascular hemolysis. These effects were seen in a broad range of treatment doses, even if more sustained inhibition was seen at the dose of 200 mg twice a day (which showed a safety profile overlapping to that of lower doses).^102^

The safety and the efficacy of iptacopan are now under investigation within large phase III trials in both PNH patients inadequately treated by C5 inhibitors (in a head‐to‐head randomization), as well as in untreated PNH patients. In both studies the selected dosing regimen was 200 mg orally, twice a day.

## 7 ACHIEVEMENTS AND PITFALLS OF ALTERNATIVE PATHWAY TARGETING THERAPIES

Different strategies of therapeutic interception of the complement alternative pathway were developed and exploited for the treatment of PNH. Emerging data with three different classes of compounds targeting either C3, FD or FD consistently demonstrate potential effectiveness irrespective of the specific target. Indeed, all proximal complement inhibitors acting upstream C5 (which are all alternative pathway inhibitors) are able to prevent the emergence of C3‐mediated extravascular hemolysis, and may improve the control of MAC‐mediated intravascular hemolysis (confirming that they also inhibit the terminal pathway, which is armed by the alternative pathway). Remarkably, the clinical results achieved with these proximal complement inhibitors were impressive, since they led even to hemoglobin normalization in several patients, and all biomarkers of disease activity were also normalized. These findings were never observed with any terminal inhibitor, where even in case of good hematological response residual disease activity is always detectable. For instance, the normalization of reticulocyte count and the increase of PNH erythrocyte population to percentage > 90% suggest that these treatment result in a normal life‐span of PNH erythrocytes. While these data represent remarkable advances in hematology and medicine in general, some questions remain open, and new ones are emerging. It is now clear that we can improve the hematological response in PNH patients, possibly aiming even to normalize hemoglobin and other biomarkers of disease, setting higher the bar of treatment goals. However, these achievements may bring some potential risks, which still need to be addressed. Indeed, inhibitors of the alternative pathway and proximal complement inhibitors are in general more efficient in disabling the complement cascade (since they act at the level of the enzymatic events which amplify the initial activation), possibly impairing its beneficial effects on protection from intruders and host damaged tissues. Based on available data, the use of proximal complement inhibitors seems not to increase the risk of infectious complications (or even affect their course), nor of auto‐immune diseases. Nevertheless, long‐term data need to be generated specifically looking at the risk of these complications, as well as at the risk of malignancies. In addition to these potential side effects, the use of these more potent therapies led to the emergence of potential complications resulting from their better efficacy; this is the case of breakthrough hemolysis, which may result from some leakage of inhibition. The detailed discussion of breakthrough hemolysis is out of the purpose of this manuscript and it was recently described elsewhere^103^; nevertheless, here we have to acknowledge that the more effective an anti‐complement treatment is, the more severe breakthrough hemolysis may appear in case of loss of inhibition. This is largely due to the fact that in PNH more effective anti‐complement treatment result in larger proportion of PNH erythrocytes, which remain susceptible to complement hemolysis. We have to highlight that in the past we have never seen such large GPI‐deficient erythrocyte population in PNH patients, and we have to learn how this unusual condition may affect the clinical course of the disease, possibly with some risk of peculiar complications. Indeed, if the protection from the hemolysis is lost for any reason (low drug plasma level – pharmacokinetic breakthrough, or brisk complement activation secondary to some trigger amplifying steady‐state complement activation – pharmacodynamics breakthrough), the extent of hemolysis would be much larger, with more severe clinical presentation. In these circumstances, which were seen for instance with pegcetacoplan^91^ and iptacopan^101^ (both leading to percentage of PNH erythrocytes >90%, demonstrating their outstanding efficacy), the pharmacological features of the specific inhibitor are crucial in driving transient loss of full inhibition possibly leading to breakthrough hemolysis.

Thus, even if the initial data with proximal complement inhibitors clearly demonstrated that they are effective even in monotherapy since they effectively disable the terminal pathway by switching off upstream steps of the complement cascade, the debate about the preference between monotherapy or combination treatment remains lively. A recent review by our masters Dr. Notaro and Prof. Luzzatto addressed for the first time at the molecular level the mechanistic reasons underlying breakthrough hemolysis in PNH with different complement inhibitors.^104^ We fully agree that the enormous amplification potential associated with enzymatic activities existing upstream the terminal pathway drives the risk and the actual presentation of breakthrough hemolysis, which seems different between terminal and proximal complement inhibitors.^103^ From one side, the blockade of the terminal complement is more frequently associated with breakthrough hemolysis, because (in addition to possible pharmacokinetic breakthrough), in absence of proximal inhibition, brisk complement activation eventually overcomes C5 inhibition due to C3b‐dependent mechanisms.^104^ On the other side, by acting upstream any enzymatic step, therapeutic inhibition of the proximal complement may better prevent the activation of the terminal pathway; but any leakage of this inhibition would eventually be amplified by downstream enzymatic reactions, leading to massive activation of the terminal pathway and subsequent breakthrough hemolysis.^103^, ^104^ Our friends Dr. Notaro and Prof. Luzzatto state that combination treatment with terminal and proximal inhibitors might be the best strategy for PNH^104^; however, clinical evidences are lacking, and unfortunately none of the recent or ongoing phase III trials is comparing proximal inhibitors in monotherapy vs their combination with terminal inhibitors. While we agree that this may be the safest approach, especially at time of brisk complement activation,^94^ we still believe that the near future of complement inhibition in PNH will be based on proximal inhibitors in monotherapy.^105^ Our group demonstrated that different proximal inhibitors (a C3‐inhibitor,^80^ a FD‐inhibitor,^85^ a FB‐inhibitor,^86^ and two FH‐based recombinant proteins)^54^, ^72^ fully inhibit the lysis of PNH erythrocytes in vitro more efficiently than eculizumab, which is rather associated with residual hemolysis (of different extent, depending on the rate of complement activation).^55^ Notably, the presence of sub‐inhibitory levels of eculizumab does not change the dose–response curve of proximal inhibitors in vitro (unpublished data and Figure 2F), suggesting that a combination treatment with terminal and proximal complement inhibitors does not have a synergistic effect, but simply an additive one (still potentially useful in the clinic, because two distinct checkpoints are unlikely to be by‐passed at the same time). The risk of breakthrough hemolysis is associated to the likelihood that an effective inhibitor may drop below its therapeutic range, and/or that external conditions (such as CAC) may overcome even a therapeutic range of a given inhibitor. Thus, the potential advantage of a combination treatment is limited to the fact that the concomitant inhibition of more complement components requires that both inhibitions are incomplete to develop breakthrough hemolysis (which likely still occurs, if none of the blockades is complete). In monotherapy, in contrast to C5‐inhibition, targeting proximal components of the complement cascade (upstream the enzymatic amplification), paradoxically, should not suffer from the impact of complement‐amplifying conditions on the pharmacokinetics and the pharmacodynamics of a given proximal inhibitor, since their targets (FD, FB, and possibly C3) are not directly affected by such conditions. However, the precise mechanism of inhibition (eg, reversible vs irreversible) and the pharmacological properties of the specific inhibitor (more than their own targets), eventually resulting in different extents of incomplete inhibition, play a major role in the severity of breakthrough hemolysis. With this regard, we should take some lessons from anti‐C5 treatment strategies: the severity of breakthrough hemolysis using the RNA‐based anti‐C5 ALN‐CC5 in monotherapy^106^ was clinically more relevant than that observed with eculizumab, even if they share the same common target C5. This observation suggests that the consequences of an incomplete blockade are not the same for all inhibitors, since some agents may still exert a clinically meaningful effect limiting the severity of breakthrough hemolysis even in presence of residual complement activity (as shown by CH50).^107^ The scenario of an incomplete blockade is still unclear with proximal inhibitors, since breakthrough hemolysis was relatively infrequent but potentially severe with the C3‐inhibitor pegcetacoplan,^91^ frequent but usually mild with the FD‐inhibitor danicopan,^95^ and apparently negligible with the FB‐inhibitor iptacopan.^101^ However, breakthrough hemolysis is not unavoidably seen when a proximal inhibitor is used in monotherapy, even in presence of complement‐amplifying condition, provided that the blockade is pharmacologically complete and the amount of target molecules (either FD, FB or C3) available for activating the enzymatic amplification steps remain negligible. As a consequence, as originally hypothesized by one of us,^51^ we still think that one proximal inhibitor may be sufficient to safely and efficiently treat PNH patients, because the risk of breakthrough hemolysis is not intrinsic to the use of proximal inhibitors in monotherapy.^105^ However, breakthrough hemolysis emerged as an essential clinical endpoint needing specific evaluation in ongoing clinical studies, and mechanistic data unraveling the actual causes of breakthrough hemolysis with each complement inhibitor are demanded. In the meantime, combination treatment may be a safe risk‐mitigation strategy (even transient, as single‐dose rescue treatment) in case of severe breakthrough hemolysis occurring during treatment with proximal inhibitors in monotherapy.

## 8 CONCLUSIONS

Several years ago one of us described C3‐mediated extravascular hemolysis as novel mechanism of disease emerging during anti‐C5 treatment for PNH, eventually proposing upstream interception of the complement cascade as possible treatment for PNH.^48^, ^50^ Since then, we have generated several experimental data supporting this hypothesis, providing evidence that anti‐complement agents targeting different steps of the complement alternative pathway may effectively protect PNH erythrocytes from complement‐mediated damage in vitro. These preclinical data paved the way for the clinical development of different proximal complement inhibitors, which were investigated in different PNH patient populations. Clinical data confirmed the initial hypothesis, since the C3‐inhibitor pegcetacoplan, the FD‐inhibitor danicopan and the FB‐inhibitor iptacopan all resulted in improved hematological benefit in PNH patients. Notably, as anticipated by our in vitro data, these agents were effective also in monotherapy, even if their pharmacological properties potentially affect their efficacy and mostly their safety in monotherapy, Indeed, breakthrough hemolysis emerged as possible adverse event during treatment with proximal inhibitors in monotherapy, and further data are needed to better understand how best use these inhibitors of the complement alternative pathway. In the meantime, once that the clinical use of modulators of the alternative pathway seems legitimize in PNH, the next challenge is now identifying other fields of medicine which might benefit from the use these agents.

## CONFLICT OF INTEREST

AMR has received research support from Alexion, Novartis, Alnylam, and Rapharma; lecture fees from Alexion, Novartis, Pfizer, and Apellis; served as a member of the advisory/investigator board for Alexion, Roche, Achillion, Novartis, Apellis, Biocryst, and Samsung; and served as consultant for Amyndas.

## Data Availability

Data sharing is not applicable to this article as no new data were created or analyzed in this study.
